# Preliminary evaluation of a robotic apparatus for the analysis of passive glenohumeral joint kinematics

**DOI:** 10.1186/1749-799X-8-24

**Published:** 2013-07-24

**Authors:** Claudio Rosso, Andreas M Müller, Vahid Entezari, William A Dow, Brett McKenzie, Stacey K Stanton, Daniel Li, Andrea Cereatti, Arun J Ramappa, Joseph P DeAngelis, Ara Nazarian, Ugo Della Croce

**Affiliations:** 1 Center for Advanced Orthopaedic Studies, Beth Israel Deaconess Medical Center and Harvard Medical School, 330 Brookline Avenue, RN115, Boston, MA 02215, USA; 2 Orthopaedic Department, University Hospital Basel, University of Basel, Basel, Switzerland; 3 Department of Orthopaedic Surgery, Beth Israel Deaconess Medical Center, Harvard Medical School, Boston, MA 02115, USA; 4 Department of Information Engineering, Political Sciences and Communication Sciences, University of Sassari, Sassari 07100, Italy

**Keywords:** Shoulder biomechanics, Shoulder range of motion, Motion analysis, Glenohumeral joint

## Abstract

**Background:**

The shoulder has the greatest range of motion of any joint in the human body. This is due, in part, to the complex interplay between the glenohumeral (GH) joint and the scapulothoracic (ST) articulation. Currently, our ability to study shoulder kinematics is limited, because existing models isolate the GH joint and rely on manual manipulation to create motion, and have low reproducibility. Similarly, most established techniques track shoulder motion discontinuously with limited accuracy.

**Methods:**

To overcome these problems, we have designed a novel system in which the shoulder girdle is studied intact, incorporating both GH and ST motions. In this system, highly reproducible trajectories are created using a robotic actuator to control the intact shoulder girdle. High-speed cameras are employed to track retroreflective bone markers continuously.

**Results:**

We evaluated this automated system’s capacity to reproducibly capture GH translation in intact and pathologic shoulder conditions. A pair of shoulders (left and right) were tested during forward elevation at baseline, with a winged scapula, and after creation of a full thickness supraspinatus tear.

**Discussion:**

The system detected differences in GH translations as small as 0.5 mm between different conditions. For each, three consecutive trials were performed and demonstrated high reproducibility and high precision.

## Background

The glenohumeral (GH) joint has the greatest range of motion (ROM) of any joint in the human body. It benefits from both the humeral head motion in the glenoid and the scapular motion against the thorax. Given the limited articular congruency of the GH joint, the shoulder joint demonstrates little intrinsic stability, enjoys a tenuous relationship between motion and stability, and is easily injured [1].

In order to better understand the mechanisms of injury and establish appropriate treatment protocols for shoulder pathology, several biomechanical models have been developed. Many techniques study the GH joint in isolation, without consideration of scapulothoracic (ST) motion or the position of the clavicle [2-7]. Other methods rely on discrete and manual manipulation of the shoulder over a limited motion trajectory [8-11]. This methodology introduces significant errors in measurement, positioning, and reproducibility. Additionally, the approach simplifies shoulder motion, thereby negating its dynamic nature.

To improve upon current techniques, we have developed an automated testing apparatus that was designed to test the intact shoulder girdle and assess its kinematics with excellent reproducibly and high precision and accuracy [12,13]. This system manipulates an intact cadaveric torso using robotic technology to reproduce patterns of motion for the upper extremity. Motion data are captured continuously using retroreflective bone markers for continuous stereophotogrammetric analysis. Therefore, the goal of this study was to assess the reproducibility of the presented automated techniques and kinematic analysis of GH translation during forward elevation of the GH joint in cadaveric tissue.

## Methods

### Testing apparatus

**Figure 1:**
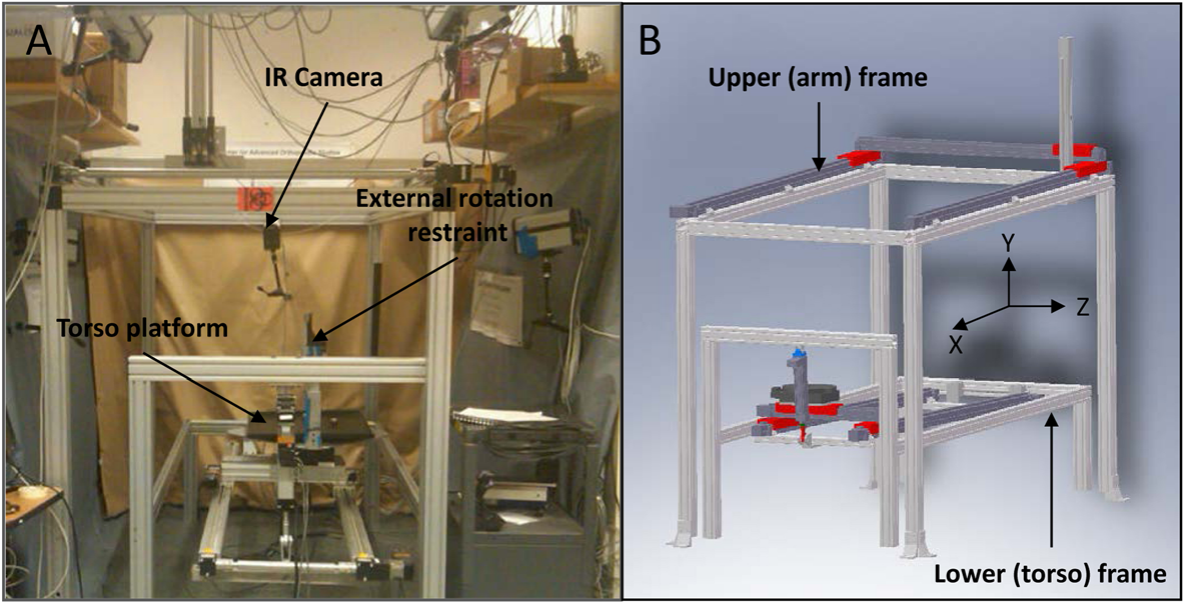
**Robotic testing system that generates automated motion segments for a cadaveric torso over a designated trajectory.** The seven degrees of freedom testing apparatus was designed and manufactured with four actuators on the lower frame to move the torso and three actuators on the upper frame to move the hand with an additional rotational axis added to the lower frame to rotate the torso. **(A)** Apparatus photograph. **(B)** Apparatus schematic.

A robotic testing system that generates automated motion segments for a cadaveric torso over a designated trajectory was designed and manufactured (Figure 1). The robotic system consists of lower (torso) and upper (hand) frames that provide linear and rotational motion along seven axes. The lower frame generates motion along *X*, *Y*, and *Z* axes and around the *Z*-axis while the upper frame generates motion along the *X*, *Y*, and *Z* axes. Motion is generated using linear and rotary closed loop actuators that are controlled via a centralized programmable system to generate any motion trajectory within the actuators’ limits. Limit and home switches are combined with encoders to produce closed loop feedback for each axis, ensuring safety and precision. The precision and accuracy of the testing system in reproducing pure and complex trajectories have been established in a separate publication [13].

A fresh frozen human torso was acquired from Medcure Anatomical Tissue Bank (Orlando, FL, USA). The torso was mounted onto a rod fixture and held in place with volume expanding foam to provide a support construct. After securing the torso to its frame, the hand was removed at the distal radioulnar joint (wrist disarticulation), and the arm was secured directly to the upper frame using a Schanz pin (Synthes, Paoli, PA, USA) inserted through the radius and the ulna. In order to test the GH translation, forward elevation motion was simulated in the sagittal, scapular, and coronal planes.

### Motion analysis

**Figure 2:**
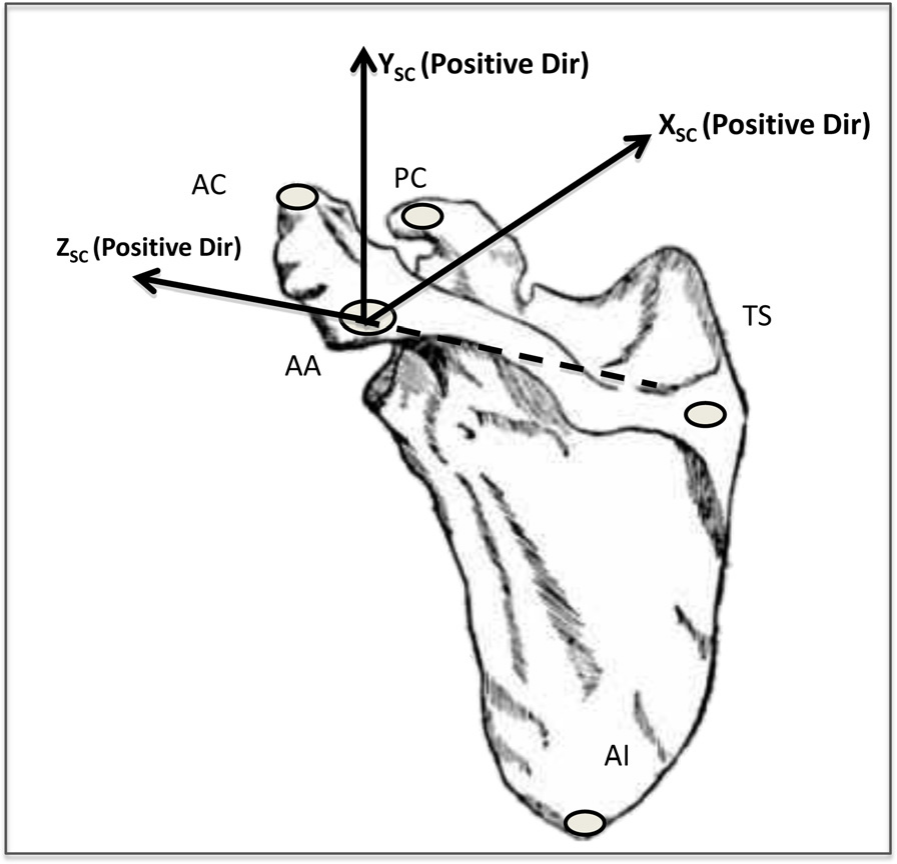
**Schematic presentation of joint coordinate system (JCS) based on anatomic landmarks defined by the ISB.** AA, acromion angle; AI, inferior angle of the scapula; TS, trigonum spinae (root of the scapular spine); PC, most ventral point of the coracoid process.

Five Qualisys ProReflex (Qualisys AB, Gothenburg, Sweden) high-speed cameras (120 frames per second) were used to record the motion of passive retroreflective, bone-embedded marker clusters (four markers/cluster for redundancy). Prior to testing, the cameras underwent a multiaspect calibration process to ensure accurate data collection, enabling them to discern motion segments as small as 0.5 mm. Bone pins equipped with marker clusters were inserted into the humerus, scapula, and thorax of the cadaver. Anatomical landmarks were calibrated with respect to the bone-embedded marker clusters using a point wand technique at locations defined by the International Society of Biomechanics (ISB) [14,15]. These data were used to create a reference coordinate system for the scapula (Figure 2). The calibrated landmarks were used to locate the center of motion for the GH joint according to Meskers et al. [16]. The translation of the GH center of motion was described using the scapular coordinate system in reference to the resting position of the arm (arm hanging along the side of the torso).

### Cadaveric proof of concept

Two shoulders from one cadaveric torso from a healthy subject with a body mass index of 35.3 and humerus length of 305 mm were tested. All motions were recorded in three trials. First, forward elevation was simulated by moving the intact arm from 0° to 160° of abduction in the coronal plane. The same motion was then repeated when the scapula was winged (reversible perturbation). The efficacy of this model of scapular winging has been illustrated in a previous investigation demonstrating its internal validity and reversibility [17]. In brief, the inferior medial angle of the scapula was elevated away from the thorax by attaching a cylindrical wedge (24 mm in height and 50 mm in diameter) to the underside of the scapula. Once secured to the bone, scapulothoracic motion was unencumbered. To restore the native state and illustrate the model reversibility, the wedge was removed and the scapula was allowed to return to its resting position. The forward and abduction motions were repeated.

The specimen was then subjected to forward elevation motion from 0° to 160° of abduction, this time in the scapular plane. After three repetitions using the intact specimen, a full thickness tear (3 cm anterior to posterior) of the supraspinatus tendon was created, and the motion was tested in this pathologic state (irreversible perturbation). The rotator cuff tear was then repaired using a transosseous-equivalent (double row) technique by board-certified orthopedic surgeons (AJR and JPD), and forward elevation was repeated. This process was repeated on the other shoulder of the cadaveric torso.

### Statistical analysis

The captured motion was divided into ten equal time intervals with the same number of frames. The average GH translations in *X*, *Y*, and *Z* axes were normalized to the baseline values of the native case in the intact shoulder case. In order to demonstrate the precision of the testing apparatus in measuring GH translations, the standard error of the mean (SEM) and intraclass correlation coefficient (ICC) for the average GH translation in *X*, *Y*, and *Z* axes were reported for scapular winging and the supraspinatus tear and repair conditions. A repeated measures ANOVA with Greenhouse-Geisser correction was used to test the difference in mean GH translation at baseline (BL), scapular winging (SW) and restored scapular (RS) positions and between BL, ST and supraspinatus repair (SR) positions. A *post hoc* analysis with Bonferroni correction was used to analyze the differences between testing conditions. Data analysis was performed using SPSS statistical software (version 21.0; IBM, Armonk, NY, USA), and statistical significance level was set at *P* value < 0.05.

## Results

During three sequential trials of forward elevation of the arm at baseline, and after winging and subsequently restoring the scapula, the GH translation was recorded with standard error of the mean of 0.02 to 0.06 mm and intraclass correlation coefficient of 0.99 to 1.00 (*P* values < 0.001) for all three axes (Table 1). Glenohumeral translation was also recorded after subjecting the arm to the same forward elevation motion at baseline and after supraspinatus tear and repair that resulted in SEM of 0.0 to 0.2 mm and ICC ranging from 0.86 to 1.00 (*P* values < 0.001) (Table 2).

**Table 1:** Baseline, scapular winging, and restored scapula

**Axis**	**Direction**	**Conditions**	**Mean**	**SD**	**SEM (95% CI)**	**ICC (95% CI)**	***P *value**
*X*	Anterior (+)/Posterior (−)	BL	2.5	1.4	0.07 (0.02 to 0.27)	0.999 (0.996 to 0.100)	0.10
		SW	2.4	1.4	0.03 (0.01 to 0.18)	0.999 (0.998 to 0.100)	
		RS	2.1	1.2	0.04 (0.01 to 0.17)	0.999 (0.998 to 0.100)	
*Y*	Superior (+)/Inferior (−)	BL	−1.4	1.2	0.03 (0.02 to 0.12)	0.999 (0.998 to 0.100)	0.32
		SW	−1.1	0.9	0.07 (0.04 to 0.41)	0.993 (0.978 to 0.998)	
		RS	−1.3	1.1	0.05 (0.04 to 0.25)	0.997 (0.992 to 0.99.9)	
*Z*	Lateral (−)/Medial(+)	BL	−4.5	2.6	0.04 (0.01 to 0.18)	0.100 (0.100 to 0.100)	<0.001^a^
		SW	−2.9	2.6	0.06 (0.01 to 0.36)	0.999 (0.998 to 0.100)	
		RS	−4.4	2.7	0.05 (0.05 to 0.12)	0.100 (0.999 to 0.100)	

^a^Statistical significance. The mean, standard deviation (SD), standard error of the mean (SEM), intraclass correlation coefficient (ICC), and its 95% confidence interval (CI) of glenohumeral translations after three trials of humeral elevation from 0° to 160° at baseline (BL) and after scapular winging (SW) and restored scapula (RS) are summarized here in this table.

**Table 2:** Baseline, supraspinatus tear, and supraspinatus repair

**Axis**		**Conditions**	**Mean**	**SD**	**SEM (95% CI)**	**ICC (95% CI)**	***P *value**
*X*	Anterior (+)/Posterior (−)	BL	0.9	1.3	0.07 (0.04 to 0.52)	0.994 (0.975 to 0.998)	0.01^a^
		ST	0.09	0.9	0.23 (0.04 to 0.86)	0.816 (0.259 to 0.954)	
		SR	2.3	2.2	0.05 (0.01 to 0.28)	0.999 (0.997 to 0.100)	
*Y*	Superior (+)/Inferior (−)	BL	0.7	1.7	0.02 (0.01 to 0.18)	0.100 (0.998 to 0.100)	0.048^a^
		ST	0.6	1.7	0.02 (0.01 to 0.07)	0.100 (0.999 to 0.100)	
		SR	−1.9	5.0	0.04 (0.01 to 0.31)	0.100 (0.999 to 0.100)	
*Z*	Lateral (−)/Medial (+)	BL	−2.5	1.9	0.04 (0.01 to 0.16)	0.100 (0.999 to 0.100)	0.001^a^
		ST	−2.6	2.1	0.02 (0.01 to 0.08)	0.100 (0.100 to 0.100)	
		SR	−5.4	1.2	0.03 (0.01 to 0.15)	0.999 (0.996 to 0.100)	

^a^Statistical significance. The mean, standard deviation (SD), standard error of the mean (SEM), intraclass correlation coefficient (ICC) and its 95% confidence interval (CI) of glenohumeral translations after three trials of humeral elevation from 0° to 160° at baseline (BL) and after supraspinatus tear (ST) and supraspinatus repair (SR) are summarized here in this table.

**Figure 3:**
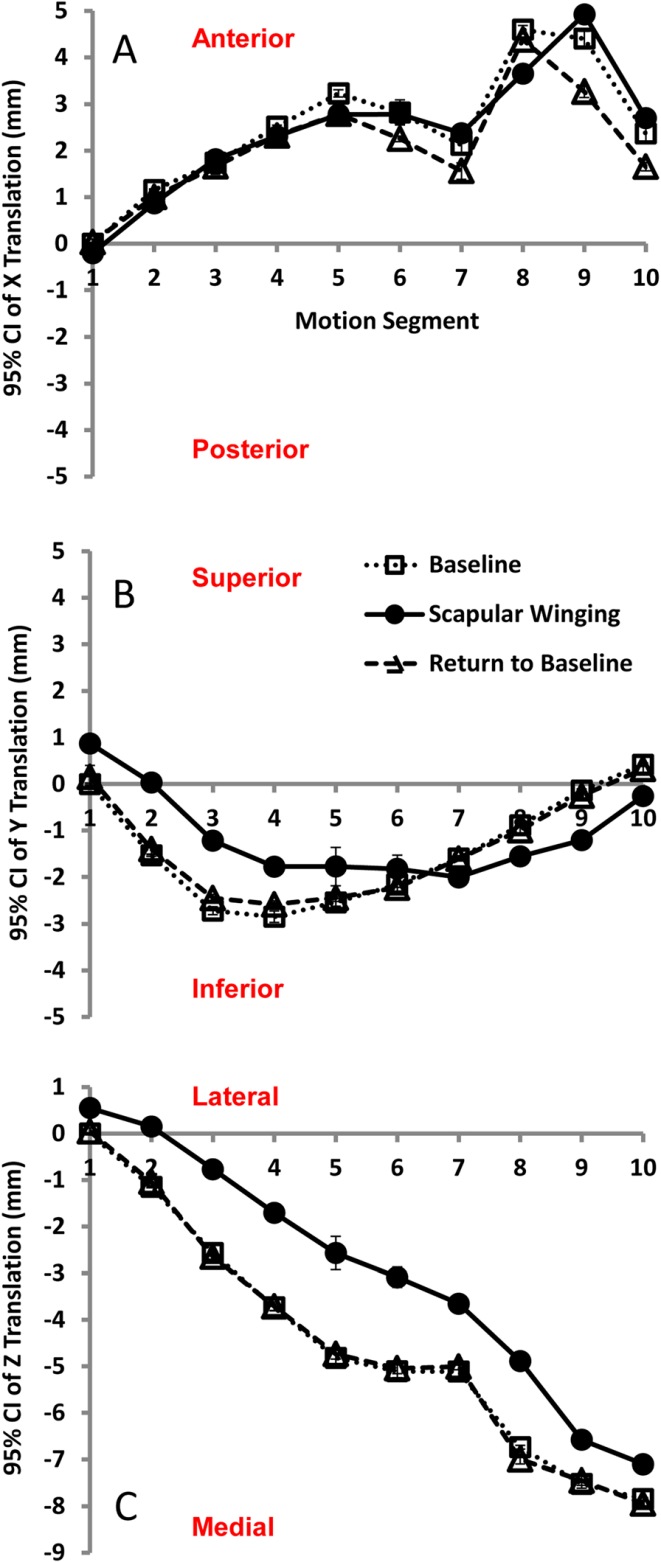
**GH translations for forward elevation following the restoration of the scapular winging. (A to C)***X*, *Y*, and *Z* glenohumeral translations obtained from the cadaveric shoulder model undergoing humeral elevation in the coronal plane at baseline, scapular winging, and restored scapular position.

The testing system was capable of discerning GH translation between baseline and reversible scapular winging conditions. Winging the scapula resulted in an average 1.6 mm medial translation (*Z*-axis) of the humeral head compared to that of the baseline (*P* < 0.001) during forward elevation of the arm (Figure 3C), while the difference was not statistically significant for *X* and *Y* axes (*P* values were 0.10 and 0.32, respectively) (Figure 3A,B,C, Table 1). As shown in Figure 3A,B,C, GH translations for forward elevation following the restoration of the scapular winging were similar to those of the baseline for all three axes.

**Figure 4:**
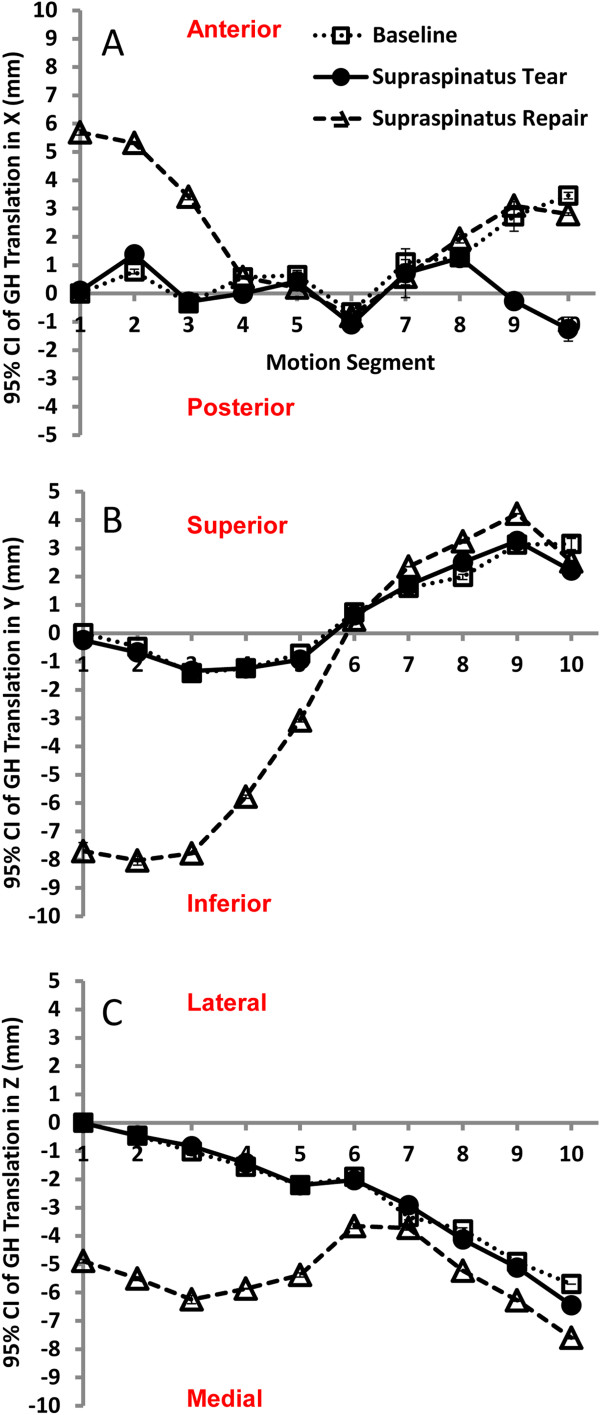
**GH translation in the 0° to 60° range of forward elevation. (A to C)***X*, *Y*, *Z* glenohumeral translations obtained from the cadaveric shoulder model undergoing humeral elevation in the scapular plane at baseline and after supraspinatus tear and repair.

The effects of supraspinatus tear and repair on GH translation are depicted in Figure 4A,B,C. During forward elevation, GH translation was not significantly different between baseline and supraspinatus tear conditions (Table 2), while supraspinatus repair on average resulted in 2.2 mm (0.2 to 4.2 mm) anterior translation, 2.5 mm (0.7 to 5.8 mm) inferior translation, and 2.9 mm (1.1 to 4.6 mm) medial translation of the GH joint in comparison to the supraspinatus tear condition (*P* values were 0.01, 0.048, and 0.001 respectively). Supraspinatus repair had a more profound effect on GH translation in the 0° to 60° range of forward elevation (Figure 4A,B,C).

## Discussion

The aim of this study was to evaluate a novel robotic system to analyze passive shoulder kinematics. To that end, we recorded GH translations during shoulder forward elevation of a cadaveric model before and after the implementation and restoration of two fundamentally different shoulder lesions that either directly involved or were distant to the GH joint. The trajectories obtained from the same cadaver at baseline and after the implementation and restoration of a specific shoulder pathology showed high reliability with an intraclass correlation coefficient of 0.82 to 1.00 and standard error of mean of 0.02 to 0.23. The system was able to detect small changes in glenohumeral translations that were caused by creation and elimination of scapular winging and supraspinatus tear and repair as two clinically relevant models.

The GH joint kinematics was affected differently by scapular winging than the torn rotator cuff. Removal of the wedge from underneath the scapula returned the pattern of motion back to its baseline. In contrast, the change associated with the torn rotator cuff was not corrected when it was repaired. This effect is seen in the overlap of the baseline and restored scapular winging trajectories, while the rotator cuff tear and repaired states are substantially different. Any inference on the efficacy of current rotator cuff repairs from a biomechanical standpoint are outside the scope of this study.

The kinematics of the shoulder joint have been studied using both *in vivo* and *ex vivo* studies. The advantage of *in vivo* investigations, as compared to our proposed system, is the contribution of active muscle forces that results in a natural motion pattern and dynamic stabilization of the GH joint. On the other hand, *in vivo* studies offer limited control and display low inter- and intrasubject reliability due to fatigue and pain [9,18-20], yet they provide accurate active loading scenarios that cannot be duplicated in ex-vivo settings. In addition, the accuracy of current approaches to track motions *in vivo* is limited. Currently, the two most common modalities, stereophotogrammetry and electromagnetic tracking, use skin markers with limited capacity to accurately record the movement of the underlying bony structures [21]. Some studies have used bone-embedded markers to overcome the error imparted by skin motion [18,20,21]. However, rigid markers in live subjects are associated with a risk of infection, change in pattern of motion due to pain, and translational/rotational instability of the actual pin [18]. Recent *in vivo* investigations have moved from tracking of markers to the use of CT and fluoroscopy for 3D tracking to 2D motion tracking, with the main limitations being radiation-exposed [22,23].

Previous *ex vivo* shoulder kinematic analyses including finite element analysis [24] and cadaveric modeling [8,11,25-29] have shown similar constraints as the proposed system with the elimination of the muscles as dynamic stabilizers of the GH joint. In some studies, loading the tendons of the selected muscles or muscle groups was simulated, but this adaptation is technically demanding for dynamic motions and is applied best on a small scale with a limited number of joints in question. Previous *ex vivo* studies have also shown high intersubject variability as observed in this study. The major improvement of this testing system over the current *ex vivo* methods is its ability to study the entire shoulder girdle (including the scapulothoracic, acromioclavicular, and sternoclavicular joints) and to reproduce a wide variety of basic and complex shoulder motions with high accuracy and precision, while allowing for real-time data acquisition. Moreover, the proposed system is capable of following an exact motion trajectory throughout all steps of the test. Nonetheless, the interaction of the apparatus, the marker clusters, and the cameras can affect the visibility of the markers and requires careful planning and operation.

There are a number of limitations associated with this preliminary study. First of all, the speed of the simulated shoulder motion was much lower than that usually observed in human subjects. This was done to avoid any unintended damage to the cadaver. While humeral elevation speed was shown to alter GH biomechanics [26] in active shoulder models, it remains unclear whether these findings can be transferred to passive cadaveric models. However, it was beyond the scope of this study to explore the association between motion speed and GH kinematics in our model. Nevertheless, this could be clarified in future studies. We included one cadaveric shoulder and demonstrated the effects of the implementation and restoration of only two shoulder pathologies with respect to the intact condition. The scapular winging condition may stand for all pathologies that are distant to the GH joint but are capable of affecting GH translations, while the rotator cuff tear may represent all pathologies that are intrinsic to the joint and can directly affect its translations. Had we not restricted this study to the analysis of these two characteristic pathologies, then the number of lesions up for debate would have easily exceeded the boundaries of an evaluation study.

Given the limited feedback on leverage forces effectively applied by the actuator to the GH joint with no dynamic stabilization of the joint by muscle forces, the extent of joint translations may vary among anthropometrically different specimens. Cadaver-specific tissue elasticity accentuated after the thawing process may further contribute to the observed interspecimen variability. There is also an inherent variability associated with the calibration of the anatomical landmarks in each specimen using a pointing wand, since these landmarks are areas rather than discrete points [29]. Nevertheless, our data indicate that changes in translation from one condition to the other may be quite consistent among different shoulders, particularly if specific segments of a motion are analyzed. Therefore, future studies may primarily compile between-condition changes observed in different specimens and subject these to paired comparative statistical tests.

## Conclusion

In conclusion, we have shown that the presented cadaveric, stereophotogrammetric testing system is capable of differentiating GH translations in sequential clinical conditions over a series of repetitions with a high degree of reproducibility in cadaveric tissue. These results confirm that this dynamic testing apparatus could be used to study cadaveric shoulder kinematics and simulate relevant clinical scenarios.

## Competing interests

The authors declare that they have no competing interests.

## Authors’ contributions

CR, AM, VE, WD, BM, SS, and DL contributed towards data acquisition, data analysis, manuscript preparation, and final approval. UD and AC contributed towards data analysis, manuscript preparation, and final approval. AR, JD, and AN contributed towards study design, data acquisition, surgical procedures, manuscript preparation, and final approval. All authors read and approved the final manuscript.
